# A Case Report on the Diagnosis of Acute Abdominal Aortic Occlusion Using Point-of-Care Ultrasound in the Emergency Department

**DOI:** 10.7759/cureus.44686

**Published:** 2023-09-04

**Authors:** Ming-Tung Wu, Craig Beringer, Zeyn Mahomed

**Affiliations:** 1 Emergency Medicine, University of Witwatersrand, Johannesburg, ZAF; 2 Emergency Department, Charlotte Maxeke Johannesburg Academic Hospital, Johannesburg, ZAF; 3 Emergency Department, Chris Hani Baragwanath Academic Hospital, Johannesburg, ZAF

**Keywords:** abdominal aortic stenosis, emergency medicine, emergency department, point of care ultrasound, acute aortic syndrome, acute aortic occlusion

## Abstract

Point-of-care ultrasound (PoCUS) can be used to detect and evaluate for an aneurysm and/or a dissection of the abdominal aorta in suspected patients in the Emergency Department (ED). Despite the routine use of PoCUS for the assessment of the abdominal aorta in suspected aortic aneurysms and dissections, there is limited literature regarding its use in the diagnosis of acute abdominal aortic occlusions in the emergency setting. This is a case demonstrating the use of PoCUS in identifying an acute aortic occlusion in a 71-year-old female patient with known hypertension and diabetes mellitus. The patient presented with central abdominal pain and bilateral lower limb weakness to the ED. The patient had multiple differential diagnoses, including a possible acute aortic occlusion of the abdominal aorta. PoCUS of the aorta was utilized to diagnose an acute abdominal aortic occlusion in the ED. The rapid diagnosis expedited the referral to vascular surgeons for definitive management. Acute abdominal aortic occlusion is a time-sensitive and life-threatening emergency. PoCUS of the abdominal aorta to detect acute abdominal occlusions allows for a rapid diagnosis with the potential to improve outcomes. A protocol for detecting acute abdominal aortic occlusion should be included in the standard aorta PoCUS scan.

## Introduction

Point-of-care ultrasound (PoCUS) has been widely utilized in emergency settings to assist with the diagnosis of multiple pathologies [1]. Over the past two decades, PoCUS has been routinely used in emergency departments (EDs) to detect abdominal aortic aneurysms and aortic dissections in suspected patients [2]. In contrast, literature on using PoCUS for diagnosing aortoiliac disease in the emergency setting is scarce.

## Case presentation

**Figure 1:**
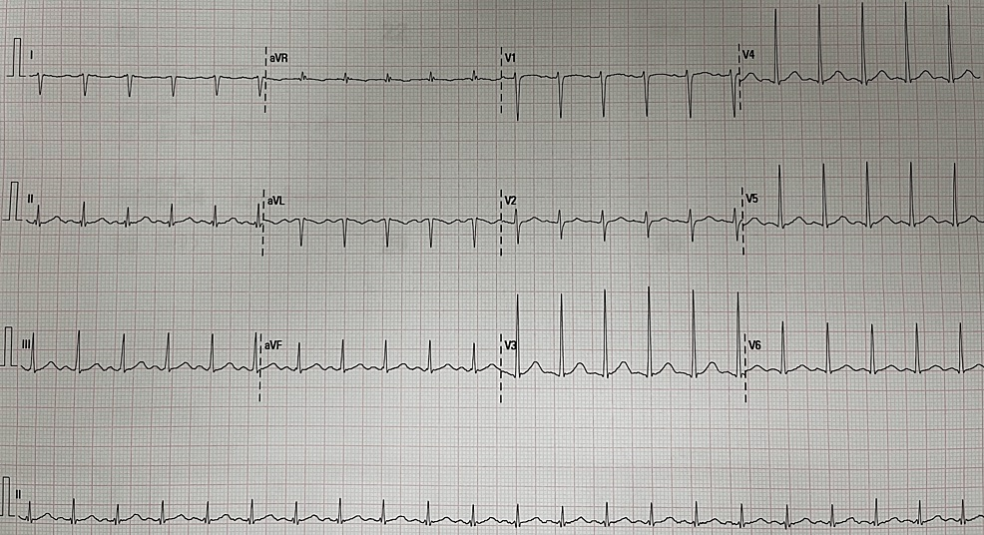
Electrocardiogram

A 71-year-old female presented to an ED at a central hospital in Johannesburg, South Africa. The patient complained of a one-day history of pelvic pain and bilateral lower limb weakness and numbness. Her vital signs on arrival included a heart rate of 142 beats per minute, a blood pressure of 118/63 mmHg, a temperature of 36.7 °C, and a respiratory rate of 25 breaths per minute. She had a background history of hypertension and diabetes mellitus. Her physical examination revealed generalized abdominal tenderness and absent bilateral lower limb pulses from the level of femoral arteries distally. Both lower limbs were cold, with associated limb paraesthesia, absent motor function, and absent sensation in the left lower limb. Her venous blood gas analysis on presentation demonstrated severe metabolic acidosis with significant hyperlactatemia and hyperglycemia (Table 1). The electrocardiogram showed sinus tachycardia (Figure 1).

**Table 1:** Venous Blood Gas

Venous Blood Gas Analysis		
Blood Gas Values		
pH	7.116	
pO_2_	31.7	mmHg
pCO_2_	33.45	mmHg
HCO_3_^-^	10.3	mmol/L
Base Excess	-17.8	mmol/L
Electrolyte Values		
K^+^	3.9	mmol/L
Na^+^	135	mmol/L
Cl^-^	116	mmol/L
Ca^2+^	1.35	mmol/L
Metabolite Values		
Glucose	25	mmol/L
Lactate	17	mmol/L

**Figure 2:**
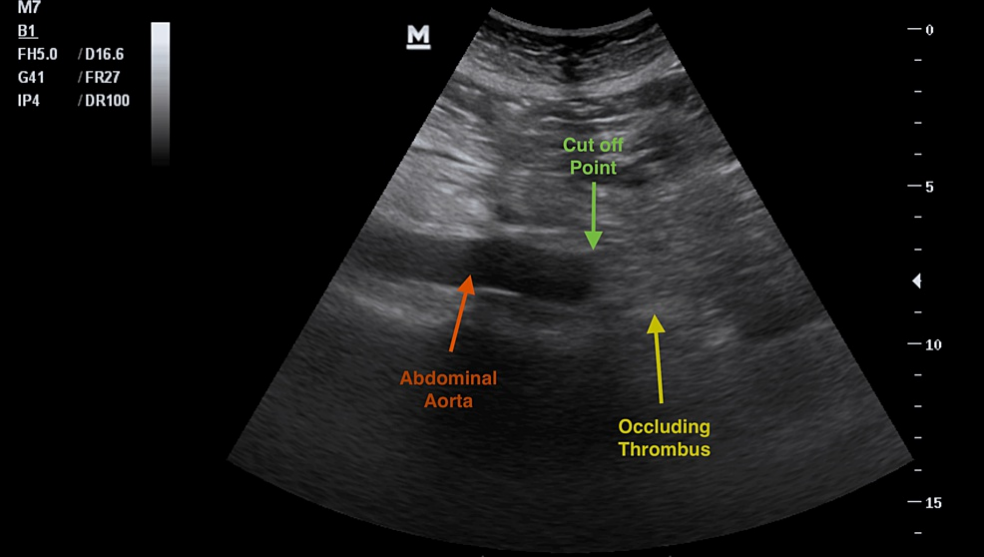
PoCUS - Longitudinal View of Abdominal Aorta PoCUS: point-of-care ultrasound

The differential diagnosis included acute spinal pathology, diabetic ketoacidosis, acute mesenteric ischemia, aortic dissection, acute limb ischemia, and acute aortic occlusion. Diabetic ketoacidosis was excluded based on a negative serum ketone level. A PoCUS of the abdominal aorta was performed in the ED, which visualized an occlusive thrombus at the distal infrarenal portion of the abdominal aorta, extending to the aortic bifurcation (Figure 2 and Video 1).

**Video 1:** Acute Occlusion at the Distal Aorta

**Figure 3:**
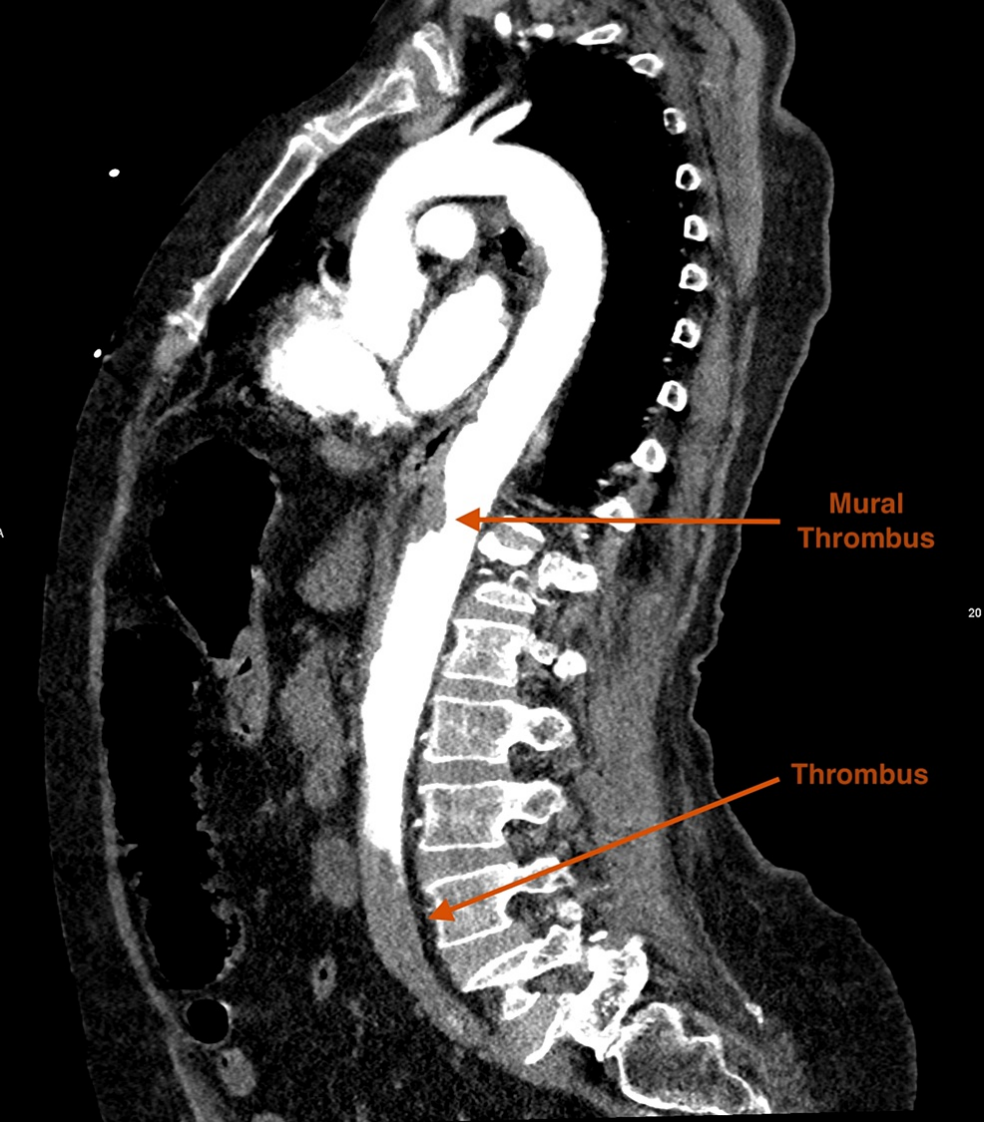
CT Angiogram - Sagittal Plane

**Figure 4:**
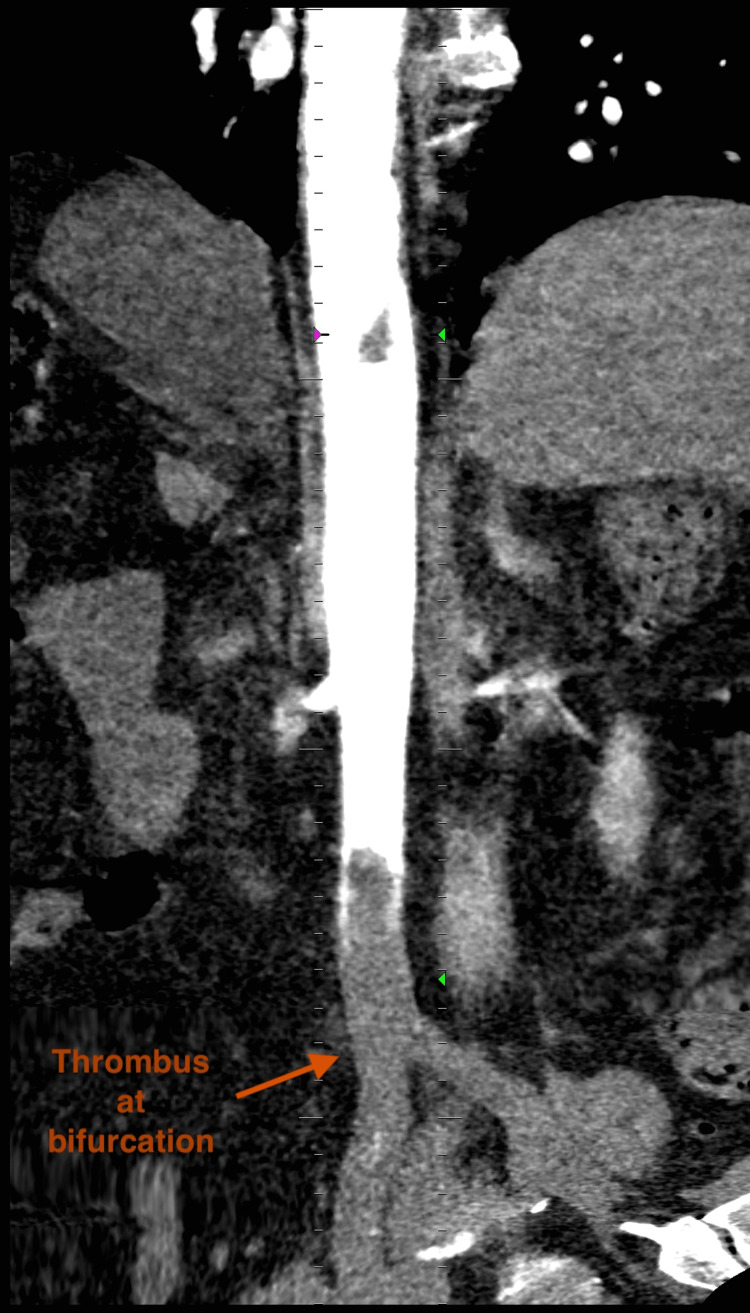
CT Angiogram - Coronal Plane

The aorta was noted to be of normal caliber, with a maximal diameter measuring 2.1 cm. Color Doppler and pulse-wave Doppler revealed absent blood flow from the level of the femoral arteries distally. The iliac arteries were not visualized due to excessive bowel gas overlying the vessels in the pelvic cavity. Transthoracic echocardiography revealed a moderately reduced left ventricular ejection fraction with no intracardiac thrombus noted. A computed tomography angiography (CTA) was performed to evaluate the spine, abdominal aorta, its branches, and distal arteries, confirming the sonographic findings of a complete distal aortoiliac occlusion. CTA demonstrated an occlusive thrombus extending from the level of L3 to the aortic bifurcation with extension into both common iliac arteries. There was an absence of contrast distal to the thrombus with no collateral blood flow as well as poor filling of the inferior mesenteric artery. Furthermore, an eccentric long-segment mural thrombus was noted on the anterior wall of the aorta extending from the level of T9 - L1. This was associated with poor to no opacification of the anterior branches of the aorta at this level (celiac trunk and superior mesenteric artery); however, no clear evidence of bowel or solid organ ischemia was noted (Figure 3 and Figure 4).

The patient was referred immediately to vascular surgery with the goal of urgent revascularization. Due to the additional CT findings, the patient was taken to the operating room immediately for laparoscopy to evaluate the extent of bowel involvement and planned to progress to an open thrombectomy if the disease state was deemed operable. Extensive bowel necrosis was identified at laparoscopy, and the patient’s condition was deemed not survivable. The patient was transferred from the operating room to the ward for palliative care due to the advanced disease state.

## Discussion

Aortic occlusion encompasses a spectrum of diseases. Patients can present acutely or with a chronic course depending on the etiology, mechanism of occlusion, the level of aorta involvement, and its branches [3]. Leriche syndrome forms a part of the spectrum of the disease and is characterized as progressive obstruction of the aortoiliac vessels. The classic patient presentation includes chronic buttock or thigh claudication and absent or decreased femoral pulses with or without erectile dysfunction [4]. Leriche syndrome should not be confused with acute aortic occlusion, which is a life-threatening emergency with high morbidity and mortality [5]. Acute aortic occlusion is rare, and the incidence ranges from 2.7 to 5 cases per 1 million per year [6]. The most common etiologies of acute aortic occlusions are large saddle embolus to the aortic bifurcation, embolism from a dislodged left ventricular thrombus, thrombosis-in-situ of the atherosclerotic aorta, and occlusion of previous surgical intervention [5,6]. Abrupt disruption in perfusion to the lower limbs alone or combined with abdominal organs determines the clinical presentation. Up to 80% of patients with acute abdominal occlusion present with bilateral acute limb ischemia, neurosensory deficit, weakness, or paralysis [7]. Pain in the abdomen and pelvis suggests a higher occlusion level and presents in up to 24% of the patients [7]. Multiple system involvement, including the bowel and kidneys, is not uncommon. The diagnosis of acute abdominal aortic occlusion can be challenging.

Patient presentation is often non-specific, with acute abdominal aortic occlusion often masquerading as many other pathologies or disease entities. Moreover, due to disease prevalence occurring in the older population with multiple co-morbidities, a delay in diagnosing acute aortic occlusion is not uncommon, considering the patient's nonspecific symptoms resulting in a broad differential diagnosis [5]. Time to revascularisation is a major determining factor in patients’ outcomes. The duration of ischemia leads to complications of the systems involved, along with the potential of proximally thrombus propagation leading to further systems involvement as time progresses [7]. Post-perfusion syndrome secondary to reperfusion injury and rhabdomyolysis has been described previously [8,9]. Prompt diagnosis decreases the duration of cellular dysfunction and necrosis, improving the time to reperfusion, and thus improving mortality and morbidity [7]. The most appropriate diagnostic test is a CTA [7], which can demonstrate the location and the extent of the occlusion. The presence of aortic atherosclerosis suggests the obstruction mechanism to be an in-situ thrombosis rather than an embolic event. Treatment methods for revascularizations depend on the etiology of the occlusion [6,7]. Interventions can be classified into the open surgery or endovascular approach. Common options include axillary-bifemoral bypass, aortoiliacal/femoral bypass, thromboembolectomy, and catheter-directed thrombolysis [6,7,10]. Incorporating PoCUS into the evaluation of the abdominal aorta occlusion expedited our patient’s management regarding further investigations and referral to the appropriate specialty, despite the poor outcome in this case.

There are several barriers to obtaining a CTA for patients with suspected acute aortic occlusion. First, time to CTA is often delayed due to failure to consider acute abdominal aortic occlusion as a differential diagnosis. Patients with acute aortic occlusion are often suffering from hemodynamic instability and are deemed too unstable to leave the ED for investigation. In South Africa, where accessibility to emergent CT is often limited, using PoCUS to confirm the diagnosis of acute abdominal aortic occlusions can expedite patients’ treatments and improve outcomes [11,12]. Using PoCUS to evaluate abdominal aortic aneurysms is required in emergency medicine and has been protocolized in assessing suspected patients. Multiple studies have demonstrated the excellent accuracy of PoCUS in evaluating aneurysms and dissections in ED settings [2,13,14]. However, due to the rarity of acute abdominal aortic occlusion, there are no formal studies or standardized protocols for using PoCUS to assess acute abdominal aortic occlusions in the ED. As such, PoCUS is not routinely used in the ED for diagnosis. Vascular surgeons and sonographers routinely utilize PoCUS to evaluate for aortoiliac disease. They evaluate all the accessible portions of the abdominal aorta using B-mode and duplex Doppler to visualize plaque, thrombus, or dissection and assess the aortic diameters. According to the Society of Vascular Sonography, the recommended time to perform a complete abdominal aortic vascular Doppler study is 48 to 71 minutes [15]. This comprehensive protocol is extensive, time-consuming, and thus unsuitable for an acute occlusion. The total duration taken by the experienced PoCUS provider to perform a focused evaluation of the abdominal aorta and detection of a thrombus through transthoracic echo in this case was just under 15 minutes. A focused protocol for acute abdominal aortic occlusion can be adapted for the ED with the use of B-mode and duplex Doppler to visualize the thrombus or embolism and estimate the level of involvement from the proximal abdominal aorta to the level of bifurcation at the common iliac vessels. The goal is to rule in abdominal aortic obstruction rather than exclude the diagnosis. The use of PoCUS in the emergency setting by a trained emergency physician for identifying acute abdominal aortic occlusion is certainly possible.

## Conclusions

Acute abdominal aortic occlusion is a time-dependent, life-threatening emergency. Diagnostic uncertainty and time to CTA impede time to revascularization. Using PoCUS to diagnose acute abdominal aortic occlusion expedites definitive treatment and can improve outcomes. Developing and incorporating an acute abdominal occlusion PoCUS protocol should become routine in EDs to improve the diagnosis of this time-sensitive disease.
